# Supplementary material for: Classification Importance of Seed Morphology and Insights on Large-Scale Climate-Driven Strophiole Size Changes in the Iberian Endemic Chasmophytic Genus Petrocoptis (Caryophyllaceae)
Source: Plants (Basel). 2024 Nov 15;13(22):3208. doi: 10.3390/plants13223208 (PMC11598283; doi:10.3390/plants13223208)
Supplement: Supplementary file 1 [file plants-13-03208-s001.zip › plants-3300602-supplementary.pdf]

Supporting information. Insights on hypothetical large scale climate-driven strophiole size changes related to water uptake in the Iberian endemic chasmophytic genus *Petrocoptis* (Caryophyllaceae).

Jorge Calvo-Yuste, Ángela L. Ruiz-Rodríguez, Brais Hermosilla, Agustí Agut, M. Montserrat Martínez-Ortega, Pablo Tejero

SUPPLEMENTARY MATERIAL AND METHODS.

Table S1. Overview of the studied *Petrocoptis* populations and their characteristics. The initial taxonomic assignment following Montserrat & Fernández Casas (1990) and morphogroup as adopted here are shown. Population code, country, location, elevation and habitat (Locality), coordinates, herbaria accession, number of individuals (N_{ind}) and number of seeds (N_{seed}) included in the morphometric analysis are indicated. The morphometric columns include the mean value of each trait per population ± standard deviation. The last row for each taxon represents the average per species (in bold).

Taxon	Population code	Morphogroup	Locality	Coordinates (WGS84)	Studied material	N_{ind}	N_{seed}	Seed width (mm)	Seed length (mm)	Seed area (mm²)	Seed roundness	Strophiole width (mm)	Strophiole length (mm)	Strophiole area (mm²)	Strophiole roundness	Strophiole relative size
P. grandiflora Rothm.	BARO	b	ES León: Carucedo, La Barosa; 551m; limestone cliff	42°29'45.4"N 6°49'17.8"W	JACAR308719	2	30	1.044 ± 0.019	1.003 ± 0.029	0.824 ± 0.010	1.044 ± 0.050	0.935 ± 0.059	0.496 ± 0.031	0.366 ± 0.047	1.894 ± 0.008	0.447 ± 0.053
	COBA	b	ES Ourense: Rubiá, Covas; 488m; limestone cliff	42°28'16.1"N 6°49'47.9"W	JACAR308717	8	45	1.159 ± 0.057	1.072 ± 0.060	0.979 ± 0.098	1.084 ± 0.026	1.051 ± 0.071	0.651 ± 0.079	0.540 ± 0.101	1.642 ± 0.125	0.552 ± 0.069
	ESTR	b	ES Ourense: Rubiá, Covas; 434m; limestone cliff	42°29'08.4"N 6°49'58.8"W	JACAR308718	7	30	1.116 ± 0.064	1.088 ± 0.060	0.956 ± 0.094	1.029 ± 0.058	1.007 ± 0.111	0.538 ± 0.073	0.435 ± 0.102	1.917 ± 0.145	0.461 ± 0.091
	OULE	b	ES Ourense: Rubiá, above Oulego; 895m; limestone cliff	42°30'45.6"N 6°55'23.3"W	JACAR308721	10	50	1.226 ± 0.053	1.131 ± 0.090	1.092 ± 0.130	1.089 ± 0.055	1.049 ± 0.095	0.617 ± 0.062	0.511 ± 0.094	1.745 ± 0.125	0.471 ± 0.074
	PENAR	b	ES León: Puente de Domingo Flórez, Peñarrubia dam; 411m; limestone cliff	42°27'19.4"N 6°49'05.8"W	JACAR308720, BIO29244, LEB18484, LEB38880, MA616247, VAL46229, VAL46808, VAL108597	8	33	1.244 ± 0.090	1.133 ± 0.061	1.110 ± 0.131	1.100 ± 0.045	1.173 ± 0.135	0.663 ± 0.080	0.638 ± 0.153	1.833 ± 0.096	0.574 ± 0.123
	SOBR	b	ES León: Sobrado, climbing area; 581m; limestone cliff	42°31'42.8"N 6°50'51.0"W	JACAR308722	3	25	1.120 ± 0.076	1.108 ± 0.104	0.979 ± 0.148	1.015 ± 0.054	0.930 ± 0.194	0.507 ± 0.127	0.386 ± 0.177	1.864 ± 0.112	0.383 ± 0.126
	VILA	b	ES Ourense: Rubiá, Vilardesilva; 616m; limestone cliff	42°27'31.3"N 6°49'31.7"W	JACAR308723	9	46	1.110 ± 0.064	1.025 ± 0.045	0.896 ± 0.083	1.085 ± 0.050	0.974 ± 0.114	0.589 ± 0.075	0.457 ± 0.102	1.674 ± 0.191	0.510 ± 0.089
								**1.146 ± 0.070**	**1.080 ± 0.050**	**0.977 ± 0.101**	**1.064 ± 0.034**	**1.017 ± 0.084**	**0.580 ± 0.068**	**0.476 ± 0.095**	**1.796 ± 0.109**	**0.485 ± 0.065**
P. pyrenaica subsp. *viscosa* Rothm.	CHAN	b	ES León: Borrenes; 613m; rocky outcrop within the forest	42°28'32.2"N 6°44'13.8"W	JACAR308728	10	49	1.123 ± 0.054	1.044 ± 0.055	0.924 ± 0.088	1.079 ± 0.029	1.029 ± 0.104	0.656 ± 0.102	0.537 ± 0.127	1.608 ± 0.208	0.579 ± 0.117
	CORN	b	ES León: Priaranza del Bierzo, castle of Cornatel; 818m; limestone cliff	42°29'15.9"N 6°41'35.8"W	JACAR308724	8	30	1.104 ± 0.097	1.017 ± 0.064	0.888 ± 0.133	1.088 ± 0.038	1.078 ± 0.076	0.590 ± 0.090	0.505 ± 0.113	1.882 ± 0.160	0.566 ± 0.040
	FERR	b	ES León: Priaranza del Bierzo, Peñas de Ferradillo; 1411m; limestone cliff	42°27'42.1"N 6°39'31.0"W	JACAR308725, JACAR308726, JACAR308727	9	47	1.030 ± 0.057	0.945 ± 0.059	0.767 ± 0.090	1.092 ± 0.032	0.862 ± 0.122	0.490 ± 0.110	0.341 ± 0.116	1.815 ± 0.229	0.447 ± 0.143
								**1.086 ± 0.049**	**1.002 ± 0.051**	**0.860 ± 0.082**	**1.086 ± 0.007**	**0.990 ± 0.113**	**0.579 ± 0.084**	**0.461 ± 0.105**	**1.768 ± 0.143**	**0.531 ± 0.073**
P. pyrenaica subsp. *glaucifolia* (Lag.) P. Monts. & Fern. Casas	ALI	b	ES Cantabria: Camaleño, above the Áliva track; 1973m; limestone rock	43°09'55.5"N 4°48'11.8"W	JACAR308832	8	41	1.159 ± 0.073	1.100 ± 0.079	1.004 ± 0.113	1.060 ± 0.073	1.107 ± 0.102	0.631 ± 0.077	0.553 ± 0.111	1.796 ± 0.138	0.548 ± 0.058
	BEJE	b	ES Cantabria: Peñarrubia, between La Hermida and Bejes; 295m; limestone rock	43°15'04.7"N 4°37'24.2"W	JACAR308849, JACAR308850, JACAR308851, HDXA12115	8	47	1.328 ± 0.098	1.215 ± 0.078	1.274 ± 0.175	1.095 ± 0.031	1.033 ± 0.141	0.716 ± 0.068	0.588 ± 0.131	1.450 ± 0.093	0.463 ± 0.082
	CARE	b	ES Asturias: Arenas de Cabrales, between Arenas and Poncebos; 189m; limestone rock	43°17'17.4"N 4°49'46.5"W	JACAR308833, VIT9174	3	29	1.254 ± 0.009	1.138 ± 0.066	1.122 ± 0.060	1.107 ± 0.069	1.020 ± 0.049	0.651 ± 0.137	0.527 ± 0.125	1.658 ± 0.384	0.474 ± 0.132
	CIGU	b	ES León: Crémenes, crossroad to Ciguera; 1151m; limestone rock	42°57'44.2"N 5°08'31.9"W	JACAR308831	8	30	1.255 ± 0.079	1.237 ± 0.069	1.222 ± 0.123	1.017 ± 0.058	1.058 ± 0.143	0.563 ± 0.099	0.471 ± 0.122	1.954 ± 0.338	0.387 ± 0.086
	COLI	b	ES Burgos: Los Altos (Colina), Ebro gorge near Quintanilla Colina; 623m; limestone rock	42°49'34.3"N 3°43'34.8"W	JACAR308842	6	30	1.122 ± 0.068	1.055 ± 0.053	0.934 ± 0.096	1.065 ± 0.042	1.172 ± 0.092	0.733 ± 0.053	0.676 ± 0.093	1.623 ± 0.089	0.726 ± 0.037
	EMIL	b	ES León: San Emiliano; 1158m; limestone rock	42°57'23.3"N 6°00'06.3"W	JACAR30844	10	50	1.009 ± 0.075	0.962 ± 0.043	0.765 ± 0.082	1.051 ± 0.054	0.919 ± 0.091	0.596 ± 0.063	0.430 ± 0.067	1.588 ± 0.190	0.563 ± 0.064
	GOVE	b	ES Asturias: Caso, Govezanes; 655m; limestone rock	43°12'19.9"N 5°21'56.2"W	JACAR308830	6	30	1.296 ± 0.091	1.191 ± 0.087	1.218 ± 0.163	1.092 ± 0.052	1.082 ± 0.162	0.585 ± 0.131	0.511 ± 0.192	1.903 ± 0.242	0.411 ± 0.112
	LINT	b	ES Cantabria: Miera, near Linto; 235m; limestone rock	43°16'44.9"N 3°42'04.9"W	JACAR308855	5	27	1.239 ± 0.066	1.226 ± 0.065	1.197 ± 0.124	1.011 ± 0.023	0.955 ± 0.047	0.650 ± 0.084	0.496 ± 0.072	1.525 ± 0.169	0.414 ± 0.056

Taxon	Population code	Morphogroup	Locality	Coordinates (WGS84)	Studied material	N_{ind}	N_{seed}	Seed width (mm)	Seed length (mm)	Seed area (mm²)	Seed roundness	Strophiole width (mm)	Strophiole length (mm)	Strophiole area (mm²)	Strophiole roundness	Strophiole relative size
	LLAN	b	ES Cantabria: Camaleño, Los Llanos, 614m; limestone rock	43°07'59.7"N 4°43'08.0"W	JACAR308834	2	30	1.329 ± 0.029	1.221 ± 0.051	1.276 ± 0.082	1.092 ± 0.023	1.169 ± 0.042	0.651 ± 0.123	0.596 ± 0.124	1.887 ± 0.231	0.468 ± 0.071
	PARM	b	ES Asturias: Teverga, Foz de Estrechura; 685m; limestone rock	43°07'01.5"N 6°03'01.5"W	JACAR 308845, VAL130840, VAL243513	6	51	1.395 ± 0.054	1.302 ± 0.035	1.428 ± 0.073	1.072 ± 0.048	1.107 ± 0.118	0.832 ± 0.038	0.719 ± 0.068	1.358 ± 0.194	0.505 ± 0.047
	PIND	c	ES Asturias: Ribadedeva, Pimiango, Cueva del Pindal; 43m; limestone rock	43°23'51.1"N 4°31'58.5"W	JACAR308848	1	7	1.441	1.322	1.500	1.092	1.300	0.795	0.820	1.645	0.542
	PONC	b	ES Asturias: Arenas de Cabrales, near Poncebos; 321m; limestone rock	43°15'05.9"N 4°50'53.0"W	JACAR308836	6	41	1.162 ± 0.077	1.053 ± 0.046	0.963 ± 0.094	1.107 ± 0.066	1.240 ± 0.073	0.722 ± 0.053	0.706 ± 0.093	1.756 ± 0.090	0.736 ± 0.085
	PONT	b	ES León: Oseja de Sajambre; 840m; limestone rock	43°07'44.9"N 5°01'38.6"W	JACAR308840, BC982080, BC982081, BIO28223, JACAR308840, LEB62689, MA32306, MA466280, MA593842, SALA94478, SANT38869, VIT54240	13	30	1.208 ± 0.078	1.179 ± 0.089	1.125 ± 0.144	1.029 ± 0.053	1.021 ± 0.154	0.607 ± 0.108	0.495 ± 0.132	1.748 ± 0.413	0.444 ± 0.119
	POTE	c	ES Cantabria: Cillorigo de Liébana, La Hermida Gorge; 236m; limestone rock	43°11'56.9"N 4°35'23.3"W	JACAR308852, LEB48229, VAL97048, VAL199921	9	30	1.371 ± 0.018	1.225 ± 0.029	1.320 ± 0.031	1.121 ± 0.034	1.326 ± 0.073	0.761 ± 0.051	0.791 ± 0.046	1.763 ± 0.21	0.603 ± 0.031
	PROA	b	ES Asturias: Proaza, Peñas Juntas gorge; 227m; limestone rock	43°13'50.0"N 6°02'09.7"W	JACAR308846	5	30	1.334 ± 0.071	1.187 ± 0.063	1.245 ± 0.116	1.126 ± 0.053	1.127 ± 0.068	0.704 ± 0.076	0.625 ± 0.103	1.688 ± 0.140	0.502 ± 0.078
	SELLA	b	ES Asturias: Ponga, los Beyos gorge; 353m; limestone rock	43°11'06.7"N 5°04'33.6"W	JACAR308839, JACAR308839, LEB22921, MA32307	8	30	1.246 ± 0.157	1.238 ± 0.134	1.225 ± 0.275	1.009 ± 0.065	1.096 ± 0.177	0.598 ± 0.137	0.526 ± 0.188	1.908 ± 0.302	0.429 ± 0.114
	SIL	b	ES León: Palacios del Sil, Cuevas del Sil; 1051m; limestone rock	42°53'48.5"N 6°23'37.9"W	JACAR308843	5	28	0.988 ± 0.056	1.013 ± 0.123	0.791 ± 0.144	0.982 ± 0.059	0.965 ± 0.091	0.550 ± 0.043	0.419 ± 0.069	1.781 ± 0.038	0.542 ± 0.110
	SOMI	b	ES Asturias: Pola de Somiedo, Somiedo; 700m; limestone rock	43°06'59.2"N 6°15'16.9"W	JACAR30853, MA618048, MA873826	5	21	1.080 ± 0.121	1.055 ± 0.098	0.903 ± 0.171	1.025 ± 0.062	1.043 ± 0.220	0.669 ± 0.057	0.547 ± 0.15	1.621 ± 0.231	0.603 ± 0.070
	TANA	b	ES Asturias: Caso, Tarna pass; 902m; limestone rock	43°07'26.1"N 5°15'02.4"W	JACAR308828	7	30	1.254 ± 0.032	1.156 ± 0.052	1.140 ± 0.063	1.089 ± 0.051	0.981 ± 0.169	0.519 ± 0.100	0.400 ± 0.091	1.988 ± 0.452	0.352 ± 0.074
	VEGA	b	ES León: Vegacervera, Vegacervera gorge; 1071m; limestone rock	42°54'11.8"N 5°32'21.0"W	JACAR308826, JACAR308827, HDXA12113	6	30	1.292 ± 0.063	1.275 ± 0.089	1.298 ± 0.149	1.017 ± 0.041	1.089 ± 0.084	0.640 ± 0.101	0.549 ± 0.098	1.765 ± 0.288	0.433 ± 0.090
								**1.238 ± 0.122**	**1.168 ± 0.099**	**1.148 ± 0.199**	**1.063 ± 0.043**	**1.091 ± 0.110**	**0.659 ± 0.083**	**0.572 ± 0.119**	**1.720 ± 0.167**	**0.507 ± 0.103**
P. pyrenaica subsp. *pyrenaica* (J. Bergeret) A. Braun ex Walp.	AGUER	b	ES Huesca: Hecho, Agüerri gully; 1713m; limestone crag	42°47'13.1"N 0°40'29.7"W	JACAR308811, JACAR308812, JACA158877, JACAV14574	5	30	1.380 ± 0.048	1.251 ± 0.062	1.357 ± 0.084	1.108 ± 0.066	1.066 ± 0.142	0.633 ± 0.047	0.537 ± 0.104	1.698 ± 0.182	0.396 ± 0.067
	AIA	b	ES Guipuzkoa: Oiartzun, Aiako Harria; 598m; granite rock	43°17'25.2"N 1°47'10.4"W	JACAR308821	5	30	1.158 ± 0.056	1.163 ± 0.112	1.059 ± 0.118	1.007 ± 0.116	0.843 ± 0.019	0.532 ± 0.075	0.353 ± 0.057	1.656 ± 0.264	0.341 ± 0.075
	ARCE	b	ES Álava: Valdegovía, Sierra de Árcena; 1249m; limestone rock	42°47'24.8"N 3°07'29.9"W	JACAR308815, JACAR308816, JACAR308817	8	47	1.379 ± 0.108	1.245 ± 0.057	1.354 ± 0.166	1.109 ± 0.042	1.185 ± 0.095	0.692 ± 0.072	0.648 ± 0.117	1.740 ± 0.126	0.482 ± 0.050
	ASPE	b	ES Huesca: Aínsa, Tortiellas; 2100m; limestone rock	42°46'04.6"N 0°33'29.8"W	JACAR308824	1	30	1.328	1.217	1.275	1.095	1.082	0.577	0.493	1.914	0.399
	AUBI	b	FR Pyrénées-Atlantiques: Béost, Col d'Aubisque; 1515m; limestone rock	42°58'26.4"N 0°19'05.7"W	JACAR308877, JACA447880	10	30	1.212 ± 0.058	1.094 ± 0.030	1.044 ± 0.06	1.110 ± 0.061	0.967 ± 0.101	0.543 ± 0.098	0.419 ± 0.119	1.865 ± 0.251	0.405 ± 0.109
	BELA	b	ES Navarra: Isaba, Belagua, Arrakobeiti; 968m; rocks near a stream	42°55'58.1"N 0°51'43.9"W	JACAR308820, JACA73465, JACA508367	10	30	1.255 ± 0.109	1.184 ± 0.050	1.169 ± 0.131	1.063 ± 0.085	0.905 ± 0.126	0.522 ± 0.023	0.372 ± 0.062	1.758 ± 0.231	0.321 ± 0.053
	BERI	b	ES Navarra: Uharte-Arakil, Uharte pass; 1300m; limestone rock	42°53'09.7"N 1°57'59.9"W	JACAR 308873, MA324334, VIT33857, VIT67803, VIT67806	10	38	1.316 ± 0.067	1.181 ± 0.061	1.222 ± 0.105	1.117 ± 0.065	0.959 ± 0.095	0.500 ± 0.066	0.377 ± 0.066	1.965 ± 0.333	0.312 ± 0.060
	COLLA	b	ES Huesca: Villanúa, western Collarada; 2030m; limestone cliffs	42°42'10.8"N 0°29'57.0"W	JACA300778	10	30	1.270 ± 0.085	1.170 ± 0.054	1.167 ± 0.084	1.091 ± 0.091	1.239 ± 0.162	0.652 ± 0.065	0.642 ± 0.138	1.918 ± 0.152	0.548 ± 0.090
	LAKO	b	ES Navarra: Isaba, Lakora peak; 1615m; granite rock	42°57'07.1"N 0°49'57.6"W	JACAR308823	6	40	1.238 ± 0.092	1.156 ± 0.112	1.127 ± 0.167	1.078 ± 0.101	0.971 ± 0.066	0.556 ± 0.108	0.431 ± 0.109	1.843 ± 0.315	0.381 ± 0.074
	LENI	b	ES Huesca: Fago, Peña Forca-Lenito; 2056m; sandstone rock	42°49'34.0"N 0°45'00.0"W	JACAR308818, JACAV71273, JACAV228371, JACAV235371	10	30	1.300 ± 0.084	1.192 ± 0.087	1.224 ± 0.166	1.092 ± 0.027	1.002 ± 0.079	0.476 ± 0.069	0.378 ± 0.077	2.231 ± 0.417	0.312 ± 0.050
	LITO	b	FR Pyrénées-Atlantiques: Béost, cirque de Litor; 1360m; limestone rock	42°57'20.8"N 0°17'25.7"W	JACAR308875, JACAR308876	6	45	1.211 ± 0.089	1.046 ± 0.093	1.001 ± 0.157	1.163 ± 0.040	0.987 ± 0.088	0.635 ± 0.053	0.496 ± 0.077	1.603 ± 0.076	0.498 ± 0.048
	LIZA	b	ES Huesca: Aragüés del Puerto, Lizara shelter; 1480m; limestone rock	42°45'40.7"N 0°37'54.5"W	JACAR308814, JACA454, JACA4658, JACA371469	9	27	1.241 ± 0.124	1.171 ± 0.099	1.146 ± 0.192	1.065 ± 0.079	0.830 ± 0.259	0.515 ± 0.136	0.357 ± 0.186	1.633 ± 0.290	0.308 ± 0.146
	MINTX	b	ES Navarra: Isaba, Mintxate gully; 824m; limestone rock	42°53'00.5"N 0°55'44.6"W	JACAR308871, BIO10539, SALA95420, VIT860	6	23	1.375 ± 0.131	1.229 ± 0.076	1.323 ± 0.131	1.135 ± 0.134	1.140 ± 0.135	0.579 ± 0.065	0.516 ± 0.098	2.022 ± 0.121	0.397 ± 0.070
	ORHI	b	ES Navarra: Ochagavía, Orhi peak; 1920m; limestone rock	42°59'09.0"N 1°00'19.5"W	JACAR308874, VAL100217, VIT33879, VIT33890, VIT68309, VIT84092, VIT87434	10	30	1.256 ± 0.043	1.138 ± 0.07	1.125 ± 0.088	1.108 ± 0.072	0.929 ± 0.082	0.484 ± 0.075	0.356 ± 0.068	1.980 ± 0.309	0.321 ± 0.074
	SARR	b	FR Pyrénées-Atlantiques: Sarrance, town; 286m; rock wall	43°03'02.4"N 0°36'05.8"W	JACAR308825, JACA3109, JACA551172, JACAR309041	10	30	1.164 ± 0.096	1.131 ± 0.063	1.036 ± 0.121	1.033 ± 0.082	0.962 ± 0.145	0.596 ± 0.136	0.464 ± 0.151	1.671 ± 0.275	0.447 ± 0.131

Taxon	Population code	Morphogroup	Locality	Coordinates (WGS84)	Studied material	N_{ind}	N_{seed}	Seed width (mm)	Seed length (mm)	Seed area (mm²)	Seed roundness	Strophiole width (mm)	Strophiole length (mm)	Strophiole area (mm²)	Strophiole roundness	Strophiole relative size
	ZURI	b	ES Huesca: Fago, Zuriza; 880m; limestone rock	42°48'28.8"N 0°49'57.9"W	JACAR308805, JACA269970, JACA430970	10	30	1.351 ± 0.094	1.295 ± 0.082	1.376 ± 0.139	1.051 ± 0.089	1.177 ± 0.271	0.601 ± 0.126	0.571 ± 0.222	2.023 ± 0.354	0.422 ± 0.160
								**1.277 ± 0.073**	**1.179 ± 0.062**	**1.188 ± 0.123**	**1.089 ± 0.039**	**1.015 ± 0.122**	**0.568 ± 0.063**	**0.463 ± 0.100**	**1.845 ± 0.176**	**0.393 ± 0.073**
P. hispanica (Willk.) Pau	BINI	c	ES Huesca: Fago, Biniés gorge; 610m; limestone rock	42°38'43.4"N 0°47'53.7"W	JACAR308802, JACAR308803	6	45	1.535 ± 0.076	1.353 ± 0.048	1.635 ± 0.132	1.136 ± 0.032	1.582 ± 0.074	0.865 ± 0.079	1.079 ± 0.130	1.890 ± 0.181	0.662 ± 0.091
	ESCA	c	ES Zaragoza: Salvatierra de Esca; 563m; limestone rock	42°39'29.6"N 1°00'56.3"W	JACAR308807 VAL107950, VAL226514	6	30	1.316 ± 0.095	1.158 ± 0.043	1.197 ± 0.092	1.140 ± 0.101	1.389 ± 0.224	0.731 ± 0.098	0.806 ± 0.230	1.940 ± 0.188	0.674 ± 0.186
	GUES	c	ES Navarra: Gallués, Güesa gorge; 643m; limestone rock	42°47'34.8"N 1°05'30.3"W	JACAR308808, JACAR308809, JACAR308810, HDXA12112	9	30	1.515 ± 0.095	1.189 ± 0.043	1.417 ± 0.095	1.279 ± 0.100	1.255 ± 0.115	0.689 ± 0.085	0.680 ± 0.109	1.885 ± 0.281	0.481 ± 0.072
	LUMB	c	ES Navarra: Lumbier, Lumbier gorge; 570m; limestone rock	42°37'35.8"N 1°18'09.2"W	JACAR308806 JACA170770, MA580851, VIT16553, VIT33860, VIT58550, HDXA12111	7	31	1.350 ± 0.022	1.284 ± 0.065	1.363 ± 0.082	1.056 ± 0.049	1.355 ± 0.187	0.849 ± 0.178	0.919 ± 0.267	1.662 ± 0.297	0.677 ± 0.193
	OROE	c	ES Huesca: Jaca, Oroel peak; 1425m; limestone conglomerate	42°31'12.7"N 0°32'16.4"W	JACAR308800, MA365076, MA935222, VIT33862	5	23	1.462 ± 0.100	1.260 ± 0.052	1.449 ± 0.132	1.164 ± 0.075	1.331 ± 0.091	0.711 ± 0.105	0.749 ± 0.154	1.931 ± 0.183	0.521 ± 0.090
	SANJ	c	ES Huesca: Jaca, San Juan de la Peña; 1140m; monastery walls	42°30'27.3"N 0°40'23.6"W	JACAR308801	6	45	1.506 ± 0.082	1.339 ± 0.094	1.588 ± 0.170	1.131 ± 0.073	1.427 ± 0.085	0.772 ± 0.050	0.869 ± 0.095	1.874 ± 0.107	0.555 ± 0.077
								**1.447 ± 0.092**	**1.264 ± 0.079**	**1.442 ± 0.158**	**1.151 ± 0.073**	**1.390 ± 0.111**	**0.770 ± 0.073**	**0.850 ± 0.140**	**1.864 ± 0.102**	**0.595 ± 0.087**
P. montserratii Fern. Casas	PENA	a	ES Huesca: Peñas de Riglos, la Peña reservoir; 541m; limestone cave	42°23'02.8"N 0°44'14.6"W	JACAR308792, JACAR308863	3	26	1.505 ± 0.017	1.486 ± 0.026	1.757 ± 0.013	1.017 ± 0.030	1.255 ± 0.011	0.792 ± 0.016	0.782 ± 0.021	1.593 ± 0.021	0.450 ± 0.009
	RIGL	c	ES Huesca: Loarre, Barranco de la Mota; 819m; limestone conglomerate	42°20'13.2"N 0°42'01.9"W	JACAR308793, JACAR308794, JACAR308795, HDXA12108	4	30	1.466 ± 0.052	1.395 ± 0.047	1.609 ± 0.105	1.052 ± 0.021	1.256 ± 0.034	0.816 ± 0.024	0.806 ± 0.041	1.547 ± 0.041	0.503 ± 0.022
	YEBR	a	ES Huesca: Yebra de Basa, Ermita de Santa Orosia rock belt; 1364m; limestone conglomerate	42°30'21.4"N 0°15'50.4"W	JACAR308788, JACAR308789, JACAR308790, HDXA12107	6	30	1.570 ± 0.102	1.523 ± 0.040	1.882 ± 0.161	1.031 ± 0.051	1.175 ± 0.053	0.766 ± 0.034	0.707 ± 0.049	1.544 ± 0.080	0.379 ± 0.026
								**1.514 ± 0.053**	**1.468 ± 0.066**	**1.749 ± 0.137**	**1.033 ± 0.018**	**1.229 ± 0.046**	**0.791 ± 0.025**	**0.765 ± 0.052**	**1.561 ± 0.027**	**0.444 ± 0.062**
P. crassifolia Rouy	ANGO	a	ES Huesca: Tella-Sin, Angonés gully cliffs; 1700m; limestone rock	42°36'44.1"N 0°08'26.2"E	JACAR308875, JACAR308786, HDXA12106	4	37	1.642 ± 0.139	1.717 ± 0.106	2.226 ± 0.312	0.956 ± 0.039	0.812 ± 0.141	0.693 ± 0.230	0.466 ± 0.179	1.405 ± 0.595	0.219 ± 0.091
	ANIS	a	ES Huesca: Fanlo, Añisclo canyon; 790m; limestone rock	42°31'52.8"N 0°05'07.6"E	JACAR308775, JACAR308776, JACAR308777, JACAR308778, HDXA12104	10	30	1.680 ± 0.148	1.745 ± 0.115	2.312 ± 0.333	0.964 ± 0.043	1.065 ± 0.139	0.820 ± 0.128	0.693 ± 0.177	1.322 ± 0.170	0.302 ± 0.075
	CARD	a	ES Huesca: Puértolas, San Vicenda, mallo Carduso; 1677m; limestone rock	42°36'19.7"N 0°03'24.1"E	JACAR308781, HDXA12105	8	30	1.589 ± 0.131	1.666 ± 0.087	2.084 ± 0.252	0.954 ± 0.068	0.858 ± 0.118	0.654 ± 0.181	0.452 ± 0.147	1.476 ± 0.520	0.216 ± 0.067
	DEVO	a	ES Huesca: Tella-Sin, las Devotas gorge; 715m; limestone rock	42°33'44.8"N 0°12'16.6"E	JACAR308773, JACAR308774	5	42	1.673 ± 0.071	1.646 ± 0.058	2.167 ± 0.167	1.016 ± 0.011	1.073 ± 0.109	0.762 ± 0.085	0.644 ± 0.107	1.427 ± 0.180	0.301 ± 0.063
	PARD	a	ES Huesca: Fanlo, Pardina rock belt; 1871m; limestone rock	42°36'55.0"N 0°01'36.9"E	JACAR308841	2	9	1.514 ± 0.014	1.732 ± 0.131	2.058 ± 0.136	0.877 ± 0.075	1.065 ± 0.004	0.775 ± 0.019	0.647 ± 0.016	1.381 ± 0.019	0.315 ± 0.029
	SERC	a	ES Huesca: Fanlo, road to Sercué; 950m; limestone rock	42°33'57.6"N 0°01'55.5"E	JACAR308780	5	30	1.757 ± 0.098	1.784 ± 0.057	2.468 ± 0.206	0.985 ± 0.032	1.200 ± 0.129	0.937 ± 0.067	0.884 ± 0.130	1.296 ± 0.144	0.364 ± 0.076
	SEST	a	ES Huesca: Puértolas, Sestral alto; 1987m; limestone rock	42°34'39.7"N 0°04'18.8"E	JACAR308784	3	12	1.651 ± 0.098	1.691 ± 0.027	2.194 ± 0.142	0.977 ± 0.056	1.016 ± 0.237	0.656 ± 0.077	0.523 ± 0.089	1.577 ± 0.481	0.237 ± 0.032
								**1.644 ± 0.076**	**1.712 ± 0.048**	**2.216 ± 0.140**	**0.961 ± 0.043**	**1.013 ± 0.134**	**0.757 ± 0.101**	**0.616 ± 0.151**	**1.412 ± 0.095**	**0.279 ± 0.056**
P. pseudoviscosa Fern. Casas	ARGO	b	ES Huesca: Campo, Argoné; 773m; limestone rock	42°26'19.2"N 0°23'14.2"E	JACAR308732, JACAR308733, HDXA12125	4	32	1.209 ± 0.061	1.295 ± 0.021	1.232 ± 0.066	0.935 ± 0.048	0.922 ± 0.158	0.604 ± 0.072	0.449 ± 0.126	1.565 ± 0.131	0.368 ± 0.087
	CAMP	b	ES Huesca: Campo, Sierra de Morillo; 648m; limestone rock	42°23'35.0"N 0°23'47.9"E	JACAR308735, LEB48153, MA409461, VAL132819	7	29	1.266 ± 0.040	1.244 ± 0.086	1.235 ± 0.06	1.026 ± 0.097	0.994 ± 0.094	0.621 ± 0.065	0.487 ± 0.075	1.637 ± 0.195	0.396 ± 0.071
	GABA	b	ES Huesca: Bisaurri, Gabás, tozal de San Chuán; 1593m; limestone rock	42°28'23.5"N 0°27'58.3"E	JACAR308729 JACAR308729	5	42	1.338 ± 0.135	1.291 ± 0.131	1.368 ± 0.271	1.040 ± 0.031	1.092 ± 0.325	0.725 ± 0.222	0.668 ± 0.309	1.607 ± 0.189	0.471 ± 0.192
	RUN	b	ES Huesca: Castejón de Sos, road to El Run; 1033m; limestone rock	42°29'20.2"N 0°27'32.3"E	JACAR308736, JACAR308737, JACAR308738, HDXA12126	4	30	1.285 ± 0.135	1.310 ± 0.126	1.332 ± 0.242	0.986 ± 0.071	1.049 ± 0.189	0.731 ± 0.101	0.596 ± 0.092	1.481 ± 0.407	0.454 ± 0.046
	VENT	b	ES Huesca: Castejón de Sos, Ventamillo gorge; 850m; limestone rock	42°28'56.0"N 0°26'54.5"E	JACAR308730, JACAR308731, VIT33873, VIT68794	6	45	1.274 ± 0.174	1.223 ± 0.132	1.241 ± 0.294	1.042 ± 0.041	1.141 ± 0.148	0.773 ± 0.085	0.699 ± 0.157	1.496 ± 0.143	0.574 ± 0.078
								**1.274 ± 0.046**	**1.273 ± 0.037**	**1.282 ± 0.064**	**1.006 ± 0.046**	**1.040 ± 0.085**	**0.691 ± 0.074**	**0.580 ± 0.110**	**1.557 ± 0.068**	**0.453 ± 0.080**
P. guarensis Fern. Casas	ALQU	c	ES Huesca: Alquézar, path to Vero river; 618m; limestone cliff	42°10'24.9"N 0°01'56.1"E	JACAR308748, JACAR308749, MA208444, MA395224, MA620232, HDXA12116	8	30	1.840 ± 0.070	1.657 ± 0.086	2.397 ± 0.190	1.114 ± 0.051	2.009 ± 0.217	1.226 ± 0.125	1.943 ± 0.376	1.656 ± 0.141	0.821 ± 0.190
	BIER	c	ES Huesca: Bierge, river Alcanadre; 580m; limestone rock wall	42°11'08.6"N 0°05'10.3"W	JACAR308754	2	30	1.606 ± 0.075	1.472 ± 0.169	1.864 ± 0.298	1.098 ± 0.075	1.769 ± 0.049	0.872 ± 0.059	1.211 ± 0.116	2.052 ± 0.096	0.667 ± 0.172
	LECI	c	ES Huesca: Bárcabo, Lecina mill; 682m; limestone rock	42°13'05.6"N 0°02'25.0"E	JACAR289844, JACAR289845, JACAR289847, JACAR289850, JACAR308750	10	30	1.683 ± 0.079	1.593 ± 0.074	2.112 ± 0.185	1.059 ± 0.034	1.756 ± 0.201	1.050 ± 0.131	1.466 ± 0.296	1.691 ± 0.225	0.698 ± 0.134
	NAYA	c	ES Huesca: Bierge, Naya castle; 875m; conglomerate cliff	42°13'53.0"N 0°06'10.6"W	JACAR308751, JACAR308752, HDXA12117	9	30	1.556 ± 0.072	1.493 ± 0.091	1.826 ± 0.182	1.046 ± 0.045	1.424 ± 0.122	0.892 ± 0.060	0.995 ± 0.086	1.612 ± 0.206	0.549 ± 0.041

Taxon	Population code	Morphogroup	Locality	Coordinates (WGS84)	Studied material	N_{ind}	N_{seed}	Seed width (mm)	Seed length (mm)	Seed area (mm²)	Seed roundness	Strophiole width (mm)	Strophiole length (mm)	Strophiole area (mm²)	Strophiole roundness	Strophiole relative size
	RODE	c	ES Huesca: Bierge, Mascún gully, Las catedrales; 750m; limestone rock	42°17'24.0"N 0°04'51.8"W	JACAR308753, MA395221, MA620232, SANT12272, SANT70034	6	30	1.688 ± 0.124	1.666 ± 0.159	2.210 ± 0.282	1.023 ± 0.109	1.794 ± 0.295	1.015 ± 0.179	1.472 ± 0.460	1.786 ± 0.081	0.671 ± 0.202
								**1.675 ± 0.108**	**1.576 ± 0.090**	**2.082 ± 0.240**	**1.068 ± 0.037**	**1.750 ± 0.210**	**1.011 ± 0.143**	**1.417 ± 0.354**	**1.759 ± 0.176**	**0.681 ± 0.097**
P. montsicciana O. Bolòs & Rivas Martínez	ANA	c	ES Huesca: Castillonroy; 320m; limestone rock	41°52'38.6"N 0°34'33.9"E	JACAR308769	2	30	1.621 ± 0.077	1.480 ± 0.042	1.888 ± 0.141	1.098 ± 0.019	1.690 ± 0.073	1.078 ± 0.050	1.432 ± 0.131	1.599 ± 0.014	0.760 ± 0.008
	BERA	a	ES Huesca: Veracruz, Beranúy; 1138m; limestone overhang	42°21'48.6"N 0°35'46.3"E	JACAR308761	6	29	1.541 ± 0.053	1.508 ± 0.057	1.826 ± 0.102	1.023 ± 0.046	1.158 ± 0.127	0.872 ± 0.082	0.801 ± 0.158	1.334 ± 0.083	0.440 ± 0.083
	CAMA	c	ES Lleida: Camarasa, dam; 369m; limestone rock	41°54'09.4"N 0°53'18.3"E	JACAR308757, JACAR308758, HDXA12119	6	30	1.613 ± 0.119	1.508 ± 0.084	1.918 ± 0.233	1.072 ± 0.044	1.724 ± 0.408	0.986 ± 0.179	1.376 ± 0.480	1.754 ± 0.221	0.736 ± 0.306
	CELL	c	ES Lleida: Camarasa, Celler gorge; 317m; limestone gorge	42°02'15.3"N 0°53'00.5"E	JACAR308756	10	51	1.576 ± 0.062	1.394 ± 0.075	1.729 ± 0.157	1.134 ± 0.035	1.678 ± 0.132	1.080 ± 0.054	1.422 ± 0.149	1.572 ± 0.124	0.829 ± 0.086
	CHIR	c	ES Huesca: Viacamp, la Font gully; 534m; limestone rock	42°06'01.8"N 0°41'04.6"E	JACAR308762, JACAR308763, JACAR308764, HDXA12120	5	30	1.628 ± 0.065	1.370 ± 0.039	1.756 ± 0.118	1.189 ± 0.024	1.594 ± 0.142	0.892 ± 0.115	1.125 ± 0.240	1.806 ± 0.110	0.642 ± 0.131
	TELL	b	ES Lleida: Tremp, Peña Teller; 1637m; limestone rock	42°18'55.2"N 0°49'19.6"E	JACAR308770, JACAR308771, JACAR308772, HDXA12121	7	30	1.530 ± 0.067	1.359 ± 0.059	1.636 ± 0.116	1.128 ± 0.057	1.231 ± 0.137	0.633 ± 0.055	0.617 ± 0.115	1.952 ± 0.098	0.379 ± 0.070
								**1.585 ± 0.042**	**1.437 ± 0.070**	**1.792 ± 0.106**	**1.107 ± 0.057**	**1.513 ± 0.251**	**0.924 ± 0.168**	**1.129 ± 0.349**	**1.67 ± 0.216**	**0.631 ± 0.183**
P. pardoi Pau	AGUA	c	ES Teruel: Aguaviva; 550m; limestone rock	40°47'53.1"N 0°10'07.8"W	JACAR308868, HDXA12124	5	42	1.423 ± 0.066	1.277 ± 0.062	1.430 ± 0.125	1.118 ± 0.039	1.526 ± 0.087	0.886 ± 0.089	1.063 ± 0.140	1.745 ± 0.169	0.747 ± 0.065
	ARCA	c	ES Teruel: Torre de las Arcas, Pinar del Molino; 726m; limestone rock	40°45'43.9"N 0°05'10.9"W	JACAR308745, HDXA12123	8	30	1.389 ± 0.093	1.329 ± 0.058	1.455 ± 0.151	1.045 ± 0.049	1.383 ± 0.234	0.805 ± 0.074	0.874 ± 0.142	1.759 ± 0.415	0.605 ± 0.107
	BALM	c	ES Castellón: Zorita del Maestrazgo, La Balma monastery; 697m; limestone rock	40°44'43.1"N 0°10'36.0"W	JACAR308739, JACAR308743, MA32303, MA208449, MA395169, MA395170, SALA11775, VAL616, VAL43424	10	30	1.409 ± 0.054	1.378 ± 0.091	1.527 ± 0.138	1.027 ± 0.063	1.379 ± 0.087	0.719 ± 0.053	0.780 ± 0.076	1.953 ± 0.219	0.516 ± 0.068
	BERG	c	ES Castellón: Zorita del Maestrazgo, Bergantes river; 526m; limestone rock	40°47'04.6"N 0°08'44.4"W	JACAR308744	3	30	1.450 ± 0.077	1.318 ± 0.053	1.504 ± 0.135	1.101 ± 0.032	1.395 ± 0.063	0.687 ± 0.042	0.757 ± 0.075	2.052 ± 0.069	0.502 ± 0.018
	LONG	c	ES Castellón: Zorita del Maestrazgo, Coves Llonges; 685m; limestone rock	40°44'50.7"N 0°11'21.0"W	JACAR308742	9	39	1.451 ± 0.065	1.297 ± 0.058	1.481 ± 0.120	1.121 ± 0.045	1.464 ± 0.071	0.821 ± 0.041	0.946 ± 0.057	1.797 ± 0.140	0.644 ± 0.060
	NEGR	c	ES Castellón: Zorita del Maestrazgo, Montnegrell; 883m; limestone rock	40°45'40.0"N 0°08'13.2"W	JACAR308741	9	47	1.482 ± 0.152	1.280 ± 0.114	1.503 ± 0.299	1.161 ± 0.037	1.479 ± 0.067	0.793 ± 0.093	0.919 ± 0.126	1.927 ± 0.235	0.632 ± 0.118
								**1.434 ± 0.034**	**1.313 ± 0.038**	**1.483 ± 0.036**	**1.096 ± 0.050**	**1.438 ± 0.061**	**0.785 ± 0.072**	**0.890 ± 0.113**	**1.872 ± 0.124**	**0.608 ± 0.090**

SUPPLEMENTARY RESULTS

Table S2. Values of indices for each partition of the dataset obtained with a number of clusters. Best number of clusters proposed by each index are indicated with asterisks (*) following the corresponding index value.

	k = 2	k = 3	k = 4	k = 5	k = 6	k = 7	k = 8	k = 9	k = 10	k = 11
**KL**	1.7266	1.8640	1.8753	1.5117	0.9334	4.3171*	0.3192	1.6821	1.4209	0.4180
**CH**	52.9738	55.2430*	54.0697	50.6452	47.3095	46.1908	41.8279	41.1595	39.4772	37.5770
**Hartigan**	35.7689	22.7370*	14.1815	10.3804	10.9597	4.2405	8.4225	5.7176	4.4168	8.7548
**CCC**	-1.1965	-0.6844	0.2172	-0.0530	0.0727	0.5717*	-0.1148	0.2161	0.1137	-0.1574
**Scott**	115.6572	244.7522*	313.6958	381.0142	473.7921	491.7073	561.2375	613.0931	649.4316	670.6795
**Marriot**	4052.1920	1960.7818*	1534.1101	1075.5450	513.2318	564.3939	322.1684	219.9305	176.1670	165.5216
**TrCovW**	4658.2274	2425.0785*	1618.7192	1033.5011	727.5997	559.0970	542.2828	395.7816	322.8050	330.2656
**TraceW**	453.8215	315.9864*	246.7288	209.5774	185.2377	162.4166	153.9390	138.5811	128.7647	121.5122
**Friedman**	14191.31	16077.22	18350.23	18654.19	20716.17	21586.30	23092.93	23790.90	24860.75	28789.44*
**Rubin**	1.6460	2.3640	3.0276	3.5643	4.0327	4.5993*	4.8526	5.3903	5.8013	6.1475
**Cindex**	0.2932*	0.3001	0.4149	0.4124	0.3545	0.4037	0.3810	0.3763	0.4499	0.3706
**DB**	1.2399	0.9567*	1.1416	1.2052	1.1824	1.1673	1.1801	1.1711	1.2322	1.1848
**Silhouette**	0.3731	0.4088*	0.3015	0.2727	0.2664	0.2668	0.2473	0.2522	0.2499	0.2392
**Duda**	0.6009	1.1148*	0.7866	1.6056	1.2597	1.2647	2.2231	1.7958	1.1286	4.4040
**Pseudot2**	21.2527	-6.2822*	6.5092	-6.7891	-4.3295	-2.0933	-12.1041	-6.6471	-1.0258	-15.4586
**Beale**	3.8507	-0.6058*	1.5683	-2.0133	-1.1496	-1.0475	-3.0035	-2.3284	-0.6084	-3.4811
**Ratkowsky**	0.4099	0.4350*	0.4049	0.3773	0.3528	0.3338	0.3142	0.3005	0.2873	0.2756
**Ball**	226.9108	105.3288*	61.6822	41.9155	30.8729	23.2024	19.2424	15.3979	12.8765	11.0466
**Ptbiserial**	0.5774	0.6420*	0.5636	0.5071	0.4662	0.4320	0.4326	0.4013	0.3928	0.3826
**Gap**	-0.3787*	-0.7534	-1.0784	-1.1484	-1.5217	-1.9240	-2.0344	-2.1001	-2.2041	-2.4055
**McClain**	0.5008*	0.6519	1.1472	1.6566	2.2176	2.7906	2.8074	3.4509	3.6768	3.9364
**Gamma**	0.6935	0.7918	0.7650	0.7642	0.7876	0.8013	0.8072	0.8209	0.8314	0.8380*
**Gplus**	132.9007	89.5743	87.2768	73.0014	53.8657	41.9877	40.3207	31.2246	27.5513	24.7906*
**Tau**	601.3586	681.2186*	568.1741	473.1036	399.4171	338.5858	337.6030	286.2453	271.6801	256.4524
**Dunn**	0.1222	0.1229	0.1591	0.1402	0.1230	0.1477	0.1413	0.1477	0.2311*	0.1934
**SDindex**	1.4088	0.9234*	1.2250	1.4825	1.3527	1.4294	1.4494	1.4182	1.5357	1.4178
**SDbw**	1.1811	0.4477	0.3776	0.4203	0.2949	0.2540	0.2398	0.2144	0.2112	0.1908*

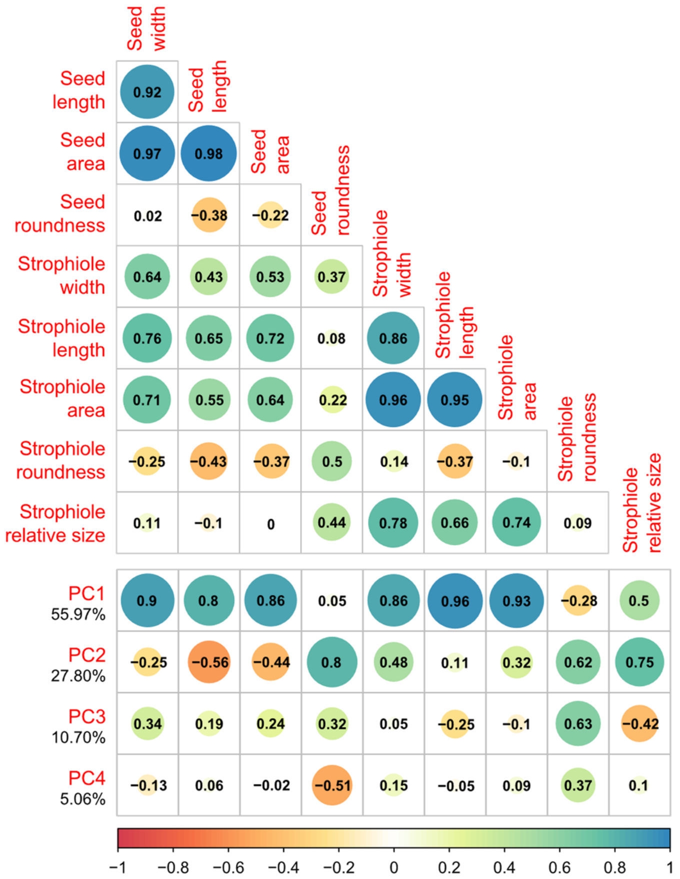

Figure S1. Variable intercorrelations (up) and correlations between variables and the first 4 principal components (down). The explained variance of each PC is also indicated.

Table S3. Confusion matrix (a), overall statistics (b) and statistics by class (c) of the model-trained random forest algorithm following the taxonomic classification of the genus into nine species (Montserrat and Fernández-Casas, 1990): *Petrocoptis grandiflora* (gra), *P. pyrenaica* (pyr), *P. hispanica* (his), *P. montserratii* (mts), *P. crassifolia* (cra), *P. pseudoviscosa* (pse), *P. guarensis* (gua), *P. montsicciana* (mon) and *P. pardoi* (par).

a)

	Reference								
Prediction	gra	pyr	his	mts	cra	pse	gua	mon	par
gra	5	11	2	0	0	2	0	0	0
pyr	67	336	17	5	0	32	0	6	18
his	1	7	12	0	0	1	1	5	6
mts	0	4	2	15	0	2	0	6	3
cra	0	0	1	5	53	1	3	6	1
pse	0	6	0	0	0	9	0	0	1
gua	0	0	2	1	0	1	28	11	1
mon	0	3	7	1	0	0	11	30	4
par	0	16	13	1	0	3	3	3	31

b)

Accuracy =	0.6314
95% CI =	(0.5974, 0.6645)
No Information Rate =	0.4659
P-Value [Acc > NIR] =	<2.2e-16
Kappa =	0.4728

c)

	gra	pyr	his	mts	cra	pse	gua	mon	par
Sensitivity	0.0685	0.8773	0.2143	0.5357	1.0000	0.1765	0.6087	0.4478	0.4769
Specificity	0.9800	0.6697	0.9726	0.9786	0.9779	0.9909	0.9794	0.9656	0.9485
Balanced Accuracy	0.5242	0.7735	0.5934	0.7571	0.9889	0.5837	0.7940	0.7067	0.7127

Table S4. Confusion matrix (a), overall statistics (b) and statistics by class (c) of the model-trained random forest algorithm following the taxonomic classification of the genus into 11 taxa, including species and subspecies (Montserrat and Fernández-Casas, 1990): *Petrocoptis grandiflora* (gra), *P. pyrenaica* subsp. *viscosa* (vis), *P. pyrenaica* subsp. *glaucifolia* (gla), *P. pyrenaica* subsp. *pyrenaica* (pyr), *P. hispanica* (his), *P. montserratii* (mts), *P. crassifolia* (cra), *P. pseudoviscosa* (pse), *P. guarensis* (gua), *P. montsicciana* (mon) and *P. pardoi* (par).

a)

	Reference										
**Prediction**	**gra**	**vis**	**gla**	**pyr**	**his**	**mts**	**cra**	**pse**	**gua**	**mon**	**par**
**gra**	21	7	17	11	3	0	0	7	0	0	0
**vis**	6	10	4	2	0	0	0	0	0	0	0
**gla**	28	20	104	48	12	3	0	16	0	3	14
**pyr**	39	2	17	74	4	0	0	6	0	3	3
**his**	1	0	5	3	14	1	0	0	1	4	8
**mts**	0	0	3	1	1	15	0	3	1	6	3
**cra**	0	0	0	0	1	5	53	2	3	6	1
**pse**	0	0	6	6	1	0	0	13	0	0	1
**gua**	0	0	0	0	1	1	0	1	28	13	0
**mon**	0	0	1	2	6	2	0	0	10	29	4
**par**	0	0	14	4	13	1	0	3	3	3	31

b)

Accuracy =	0.4769
95% CI =	(0.4423, 0.5117)
No Information Rate =	0.2348
P-Value [Acc > NIR] =	<2.2e-16
Kappa =	0.3920

c)

	**gra**	**vis**	**gla**	**pyr**	**his**	**mts**	**cra**	**pse**	**gua**	**mon**	**par**
Sensitivity	0.2877	0.2564	0.5389	0.4901	0.2500	0.5357	1.0000	0.2549	0.6087	0.4328	0.4769
Specificity	0.9399	0.9847	0.7711	0.8897	0.9700	0.9773	0.9766	0.9818	0.9794	0.9669	0.9458
Balanced Accuracy	0.6138	0.6205	0.6550	0.6899	0.6100	0.7565	0.9883	0.6184	0.7940	0.6999	0.7114

Table S5. Confusion matrix (a), overall statistics (b) and statistics by class (c) of the model-trained random forest algorithm following the taxonomic classification of the genus into five species (Walters, 1993): *Petrocoptis grandiflora* (gra), *P. pyrenaica* (pyr), *P. hispanica* (his), *P. crassifolia* (cra) and *P. pardoi* (par).

a)

Prediction	Reference: gra	pyr	his	cra	par
gra	6	11	3	0	0
pyr	66	333	43	4	21
his	1	19	23	1	8
cra	0	3	6	70	18
par	0	17	32	6	131

b)

Accuracy =	0.6849
95% CI =	(0.6519, 0.7166)
No Information Rate =	0.4659
P-Value [Acc > NIR] =	<2.2e-16
Kappa =	0.5257

c)

	gra	pyr	his	cra	par
Sensitivity	0.0822	0.8695	0.2149	0.8642	0.7360
Specificity	0.9813	0.6948	0.9594	0.9636	0.9146
Balanced Accuracy	0.5317	0.7821	0.5872	0.9139	0.8253

Table S6. Confusion matrix (a), overall statistics (b) and statistics by class (c) of the model-trained random forest algorithm following the taxonomic classification of the genus into seven taxa, including species and subspecies (Walters, 1993): *Petrocoptis grandiflora* (gra), *P. pyrenaica* subsp. *viscosa* (vis), *P. pyrenaica* subsp. *glaucifolia* (gla), *P. pyrenaica* subsp. *pyrenaica* (pyr), *P. hispanica* (his), *P. crassifolia* (cra) and *P. pardoi* (par).

a)

	Reference						
**Prediction**	**gra**	**vis**	**gla**	**pyr**	**his**	**cra**	**par**
**gra**	22	7	17	10	10	0	1
**vis**	6	12	3	2	0	0	0
**gla**	27	17	103	41	23	3	12
**pyr**	17	2	38	81	11	0	6
**his**	1	1	16	10	26	2	11
**cra**	0	0	2	1	6	69	19
**par**	0	0	14	6	31	7	129

b)

Accuracy =	0.5377
95% CI =	(0.5029, 0.5722)
No Information Rate =	0.2348
P-Value [Acc > NIR] =	<2.2e-16
Kappa =	0.4367

c)

	**gra**	**vis**	**gla**	**pyr**	**his**	**cra**	**par**
Sensitivity	0.3014	0.3077	0.5337	0.5364	0.2430	0.8518	0.7247
Specificity	0.9399	0.9859	0.8045	0.8897	0.9427	0.9622	0.9099
Balanced Accuracy	0.6206	0.6468	0.6691	0.7131	0.5928	0.9070	0.8173

Table S7. Confusion matrix (a), overall statistics (b) and statistics by class (c) of the model-trained random forest algorithm following the taxonomic classification of the genus into four species (Mayol and Rosselló, 1999; corr. Mayol and Rosselló, 2000): *Silene laxipruinosa* (lax), *S. pyrenaica* (pyr), *S. montserratii* (mts), *S. pardoi* (par).

a)

Prediction	Reference: lax	pyr	mts	par
lax	5	10	0	0
pyr	68	443	6	45
mts	0	6	69	18
par	0	31	6	115

b)

Accuracy =	0.7689
95% CI =	(0.7385, 0.7973)
No Information Rate =	0.5961
P-Value [Acc > NIR] =	<2.2e-16
Kappa =	0.5717

c)

	lax	pyr	mts	par
Sensitivity	0.0685	0.9041	0.8518	0.6461
Specificity	0.9866	0.6416	0.9676	0.9425
Balanced Accuracy	0.5276	0.7728	0.9097	0.7943

Table S8. Confusion matrix (a), overall statistics (b) and statistics by class (c) of the model-trained random forest algorithm following the taxonomic classification of the genus into seven taxa, including species and subspecies (Mayol and Rosselló, 1999; corr. Mayol and Rosselló, 2000): *Silene laxipruinosa* (lax), *S. pyrenaica* subsp. *pyrenaica* (pyr), *S. pyrenaica* subsp. *pseudoviscosa* (pse), *S. montserratii* subsp. *montserratii* (mts), *S. montserratii* subsp. *crassifolia* (cra), *S. pardoi* subsp. *guarensis* (gua) and *S. pardoi* subsp. *pardoi* (par).

a)

	**Reference**						
**Prediction**	**lax**	**pyr**	**pse**	**mts**	**cra**	**gua**	**par**
**lax**	7	11	2	0	0	0	0
**pyr**	66	385	32	5	0	1	39
**pse**	0	5	11	0	0	0	0
**mts**	0	3	1	12	0	1	8
**cra**	0	1	0	5	53	3	7
**gua**	0	1	1	2	0	26	8
**par**	0	33	4	4	0	15	70

b)

Accuracy =	0.6861
95% CI =	(0.6532, 0.7177)
No Information Rate =	0.5341
P-Value [Acc > NIR] =	<2.2e-16
Kappa =	0.4937

c)

	**lax**	**pyr**	**pse**	**mts**	**cra**	**gua**	**par**
Sensitivity	0.0959	0.8770	0.2157	0.4286	1.0000	0.5652	0.5303
Specificity	0.9826	0.6266	0.9935	0.9836	0.9792	0.9845	0.9188
Balanced Accuracy	0.5393	0.7518	0.6046	0.7061	0.9896	0.7749	0.7246
